# LION LBD: a literature-based discovery system for cancer biology

**DOI:** 10.1093/bioinformatics/bty845

**Published:** 2018-10-09

**Authors:** Sampo Pyysalo, Simon Baker, Imran Ali, Stefan Haselwimmer, Tejas Shah, Andrew Young, Yufan Guo, Johan Högberg, Ulla Stenius, Masashi Narita, Anna Korhonen

**Affiliations:** 1 Language Technology Lab, Department of Theoretical and Applied Linguistics, University of Cambridge, Cambridge, UK; 2 Institute of Environmental Medicine, Karolinska Institutet, Stockholm, Sweden; 3 Cancer Research UK Cambridge Institute, University of Cambridge, Li Ka Shing Centre, Cambridge, UK

## Abstract

**Motivation:**

The overwhelming size and rapid growth of the biomedical literature make it impossible for scientists to read all studies related to their work, potentially leading to missed connections and wasted time and resources. Literature-based discovery (LBD) aims to alleviate these issues by identifying implicit links between disjoint parts of the literature. While LBD has been studied in depth since its introduction three decades ago, there has been limited work making use of recent advances in biomedical text processing methods in LBD.

**Results:**

We present LION LBD, a literature-based discovery system that enables researchers to navigate published information and supports hypothesis generation and testing. The system is built with a particular focus on the molecular biology of cancer using state-of-the-art machine learning and natural language processing methods, including named entity recognition and grounding to domain ontologies covering a wide range of entity types and a novel approach to detecting references to the hallmarks of cancer in text. LION LBD implements a broad selection of co-occurrence based metrics for analyzing the strength of entity associations, and its design allows real-time search to discover indirect associations between entities in a database of tens of millions of publications while preserving the ability of users to explore each mention in its original context in the literature. Evaluations of the system demonstrate its ability to identify undiscovered links and rank relevant concepts highly among potential connections.

**Availability and implementation:**

The LION LBD system is available via a web-based user interface and a programmable API, and all components of the system are made available under open licenses from the project home page http://lbd.lionproject.net.

**Supplementary information:**

Supplementary data are available at *Bioinformatics* online.

## 1 Introduction

The enormous size and exponential growth of the scientific literature make it increasingly difficult for researchers to stay up to date on all developments in their field, let alone on those in related areas of study (Simpson and Demner-Fushman, 2012). This issue is particularly challenging in complex and tightly interconnected areas of biomedical research such as cancer, which is addressed in millions of existing publications. In the last two decades, there have been extensive efforts to address these challenges through the application of machine learning, natural language processing (NLP) and text mining methods to automate the processing of the biomedical scientific literature.

Literature-based discovery (LBD), first introduced and developed by Swanson in a series of seminal papers (Swanson, 1986a, 1987, 1988), seeks to uncover *undiscovered public knowledge* (Swanson, 1986b) by connecting pieces of information from disjoint literatures. The key idea behind the original LBD formulation is that concepts that are never explicitly associated in the literature may be implicitly linked through intermediate concepts in disconnected subsets of that literature. For example, Swanson (1988) found that while the literatures concerning migraine and magnesium were (nearly) isolated, they were indirectly connected through a number of concepts common to both, such as spreading cortical depression. Following Swanson’s work, LBD approaches are now commonly divided into *open discovery* and *closed discovery.* The former starts with a single concept of interest and aims to recognize potential indirectly associated concepts (hypothesis generation), while the latter assumes known start and end points and seeks to identify the most promising ways the two can be linked (hypothesis testing).

Since its inception as a largely manual exploratory process, LBD has been formalized and automated in a number of online systems such as Arrowsmith (Swanson and Smalheiser, 1997), BITOLA (Hristovski *et al.*, 2005) and FACTA (Tsuruoka *et al.*, 2008), and aspects of LBD system design and evaluation explored in a range of domain studies (Preiss *et al.*, 2012; Weeber *et al.*, 2005; Yetisgen-Yildiz and Pratt, 2008). Early LBD systems worked directly on the surface forms of words and thus lacked any way to account for the ambiguity and variability of language and biomedical terminology (Lindsay and Gordon, 1999). Such approaches necessarily miss some connections and generate other spurious ones: on one hand, they have no way to identify that e.g. *p53* and *TP53* refer to closely associated entities; on the other, they cannot determine whether e.g. *DBP* refers to *diastolic blood pressure* or *D site binding protein.* A number of more recent systems have incorporated NLP methods to map from words to concepts, most commonly using the MetaMap tool (Aronson, 2001) to map to the UMLS terminology (Bodenreider, 2004). While dictionary- and rule-based systems such as MetaMap can offer broad coverage of domain concepts, their accuracy in recognizing and disambiguating names of specific biomedical entities falls notably behind that of more recent machine learning-based methods. The recognition of biomedical entity names and their *grounding* (or normalization) to specific database or ontology identifiers has been a major focus of the biomedical natural language processing community for more than a decade, and a wealth of resources and methods targeting entities such as genes, proteins, drugs, chemicals and diseases have been introduced (Doğan *et al.*, 2014; Krallinger *et al.*, 2015; Kim *et al.*, 2004; Smith *et al.*, 2008; Wei *et al.*, 2016). Although machine learning methods trained on manually annotated resources are well established as outperforming other approaches in the recognition of biomedical entity mentions in text, there has been very limited application of these technologies in LBD systems to date.

In this work, we bring together state-of-the-art methods for biomedical entity recognition and literature-based discovery to create an LBD system realizing the opportunities of both lines of study. The system scales to cover the entire available literature and is built on open data, open standards and open source technologies. It supports both closed discovery and open discovery queries over very large graphs while remaining responsive in interactive use and allows users to ‘drill down’ to the source literature supporting candidate discoveries. In the text analysis, we focus on the recognition and comprehensive grounding of mentions of entities such as genes, proteins and chemicals as well as hallmarks of cancer (Hanahan and Weinberg, 2000), thus assuring coverage of concepts relevant to the molecular basis of cancer. Cancer is a complex and as of yet incompletely understood class of diseases that are the second leading cause of death and involve a large number of chemical and biomolecular entities, reactions and pathways. These are directly addressed in a massive and fast-growing subset of the biomedical research literature that is further intertwined with other biomolecular research in ways that make comprehensive manual analysis impossible. The public health implications, biomolecular complexity and the scope of the associated literature make cancer an important and potentially highly fruitful application area for LBD. To support this and other applications of LBD to the domain, we additionally introduce a new LBD evaluation dataset focused on cancer research discoveries.

## 2 Approach

The following sections detail the approach taken in the design and implementation of the LION LBD system.

### 2.1 Task setting

**Fig. 1.**
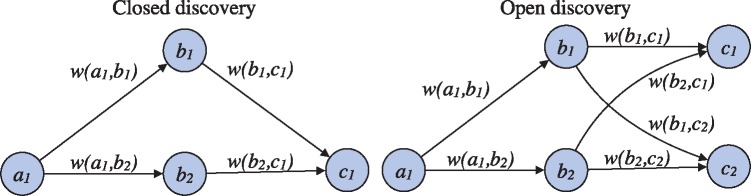
Illustration of closed and open discovery settings. In closed discovery, the goal is to identify nodes (*b*_1_, *b*_2_,…) connecting a given start and end node (*a*_1_ and *c*_1_). In open discovery, only a start node (*a*_1_) is given, and the aim is to find indirectly connected nodes (*c*_1_, *c*_2_,…). Identified candidate nodes are ranked based on the edge weights *w*

Open and closed discovery can both be defined in terms of search in graphs where nodes represent relevant domain concepts and weighted edges the strength of association between these concepts in literature (Fig. 1). We consider simple weighted graphs G=(N,E) where N is the set of nodes and E the set of edges E⊂N×N and each edge (i,j)∈E has a weight (w(i,j)). In closed discovery, given nodes a,c∈N the goal is to identify the set of nodes B⊂N such that (a,b),(b,c)∈E for each b∈B and assign each such node a score based on the weights of the edges (w(a,b),w(b,c)) on the path (*a*, *b*, *c*). We term the functions that score a path based on its edge weights *aggregation functions* and denote them by $f_{\text{g}}$. In open discovery, given a node a∈N, the goal is to identify nodes C⊂N such that (a,c)∉E and there exists a b∈N:(a,b),(b,c)∈E for each c∈C and assign each *c* a score based on the weights of edges on all paths connecting *a* to *c.* We assume with some loss of generality that the score is based on an *accumulation function*$f_{\text{c}}$ over the values of an aggregation function $f_{\text{g}}$ for each such path. The algorithmic implementation of these tasks is detailed in Supplementary Information.

The general formulation above can be parameterized through the edge weight functions w(i,j), the aggregation function $f_{\text{g}}$ and the accumulation function $f_{\text{c}}$ to realize a variety of specific open discovery and closed discovery methods. As a simple example, the edge weight can be defined as co-occurrence count and both $f_{\text{g}}$ and $f_{\text{c}}$ as sum, giving a method that prioritizes the total frequency of co-occurrence in ranking candidates. If we then assume that $w(a_{1},b_{1})=10$, other edge weights shown in Fig. 1 and there are no other (non-zero) weights, then for closed discovery the score of *b*_1_ is $w(a_{1},b_{1})+w(b_{1},c_{1})=10+2$ and that of *b*_2_ is $w(a_{1},b_{2})+w(b_{2},c_{1})=2+2$. For open discovery, the score of *c*_1_ is $w(a_{1},b_{1})+w(b_{1},c_{1})+w(a_{1},b_{2})+w(b_{2},c_{1})=10+2+2+2$, and similarly for *c*_2_. A substantial number of variants for edge weighting and scoring have been proposed in the literature, and we implement and evaluate a number of prominent alternatives in the LION LBD system (see Section 3.3). We further note that the above formulation of discovery tasks for paths of length two (one intermediate node) can be extended straightforwardly to longer paths with several intermediate nodes. However, due to the exponential growth of complexity and diminishing practical returns, these more general LBD settings are rarely used and not considered in the current implementation of the LION LBD system.

### 2.2 System architecture

The differentiation between *mention* and *entity* levels of data is central to our approach and key to allowing the LION LBD system to be both comprehensive in its coverage and computationally feasible for interactive use. On the mention level, we separately store each instance in the literature where any entity of interest is mentioned, including e.g. 270 000 mentions of *p53.* However, for the purposes of search and ranking, we formulate the graph in terms of the real-world entities that the mentions refer to, represented by their identifiers in relevant databases and ontologies. For example, *p53* is represented by the graph by a single node with the Protein Ontology identifier PR:000003035. This two-level design is also applied to the relations representing associations between concepts in the system. While in the entity-level graph there is a single edge between the node representing *p53* and the node representing *cancer*, to allow users to drill down to the evidence supporting this association, we also store and index each of the over 100 000 instances in which mentions of these entities are found together in the literature.

**Fig. 2.**
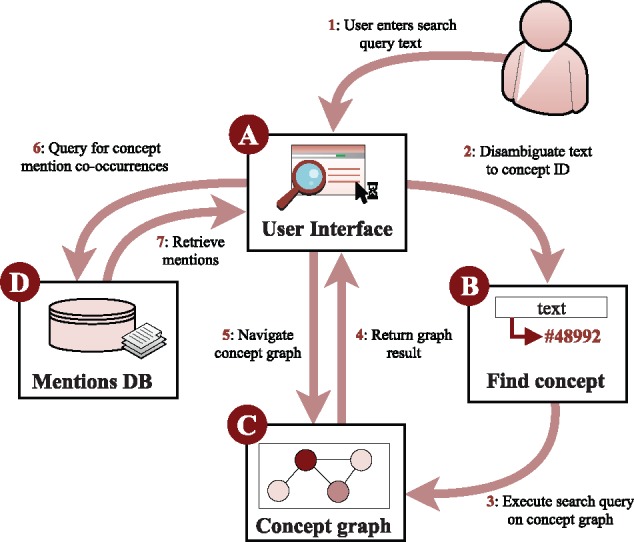
LION LBD system components. Users interact with the system through text-based queries (**A**) that are mapped to ontology identifiers (**B**) used to search the entity-level graph (**C**). Mentions of entities in context can be retrieved from a separate database (**D**)

The graph search components of the system operate solely on the basis of database and ontology identifiers, but it is necessary to allow users to query the graph using natural language strings such as *p53* and *cancer.* To implement this feature, we analyze the grounded annotations to determine the identifiers commonly associated with each string in text. For example, finding that *p53*, *TP53* and *Trp53* are frequently grounded to PR:000003035 in source data, we create a mapping from the strings to that identifier. To help resolve ambiguous cases (e.g. the species and disease senses of *Salmonella*), users are presented with alternative strings mapping to each applicable identifier (e.g. *Salmonella enterica* and *Salmonella infection*). This data is also used to determine the strings to display on the web interface to identify each node in the graph. The LION LBD system components are illustrated in Figure 2.

## 3 Materials and methods

### 3.1 Text and annotations

**Fig. 3.**
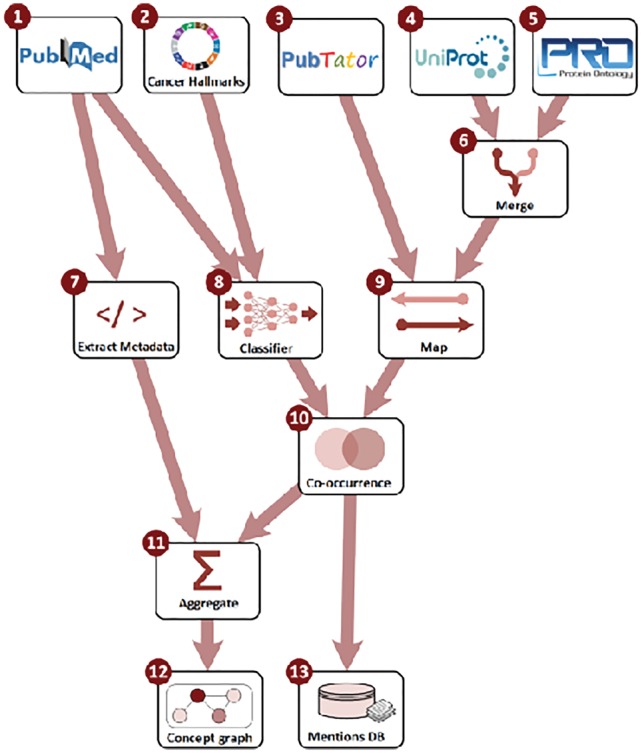
LION LBD system build process. Source data (1–5) is processed by creating a merged identifier mapping (6), metadata extraction (7), text classification (8) and identifier mapping (9). Following mention co-occurrence analysis (10), entity-level data and metrics are aggregated from mention-level data (11) and the two layers of information stored in separate databases (12 and 13)

The literature that the current release of the LION LBD system builds on is retrieved from PubMed (http://pubmed.com) and covers all of the nearly 27 million citations (titles and abstracts) in PubMed at the time of data import. We draw our annotations for physical biomedical entities, mutations and diseases from PubTator (Wei *et al.*, 2013), an online annotation resource building on state-of-the-art methods for named entity recognition and grounding. PubTator is unique among available literature-scale biomedical NLP resources in terms of its coverage of entity types and their comprehensive grounding to relevant domain databases: genes and proteins are assigned identifiers from the NCBI Gene database (Maglott *et al.*, 2004), diseases and chemicals identifiers in MeSH (Lipscomb, 2000) and ChEBI (Degtyarenko *et al.*, 2007), NCBI Taxonomy (Federhen, 2012) is used for species names, and SNP identifiers for single-nucleotide polymorphism mutations. We refer to Wei *et al.* (2013) for detailed descriptions of the methods applied for entity recognition and grounding in PubTator. To further account for cancer-related processes, we apply a dedicated machine learning system to categorize each sentence in the dataset according to the hallmarks of cancer (HoC) taxonomy of Baker *et al.* (2016, 2017), a 37-category hierarchical extension of the well-established cancer hallmarks of Hanahan and Weinberg (2000, 2011). The system classifies each sentence into zero or more of the 37 hallmark categories using a convolutional neural network. Baker and Korhonen (2017) detail the architecture of this system as well as its training and evaluation. We convert the annotations from PubTator and the HoC classifier into the uniform Web Annotation linked data representation (Sanderson *et al.*, 2017) using the JSON for Linked Data serialization (WWW Consortium, 2014) for combination and further processing using a custom pipeline implemented in Python. The choice of JSON-LD allows us to consistently use a single, standard linked data representation in all components of the system. The primary steps of the processing pipeline are shown in Figure 3. We note that the data the system is initialized with is its only major domain dependency, and the LION LBD system is already applicable to biomolecular LBD tasks in general and can be readily adapted to specifically target domains other than cancer.

### 3.2 Identifier mapping

In evaluation of early development versions of the system it was found that the granularity of the gene and protein identifiers assigned by grounding methods did not match user expectations. In particular, systems developed to address the popular BioCreative community challenge setting (Morgan *et al.*, 2008) assign NCBI Gene database identifiers to ground gene and protein mentions, thus differentiating e.g. between homologous genes in different species. While this level of detail is valuable in many applications, it was found to produce apparently redundant results in the LBD system: for example, the genes closely associated with *apoptosis* would separately include *Homo sapiens TP53*, *Mus musculus Trp53*, *Rattus norvegicus Tp53*, etc.

To address this issue, we chose to map the source NCBI Gene identifiers to the relevant identifiers in Protein Ontology (PRO) (Natale *et al.*, 2014) and then use the hierarchical structure of PRO to generalize the identifiers to a level that abstracts over homologs (*Gene level* in PRO terms). This mapping was initially implemented using the resources introduced by Huang *et al.* (2011) to map from NCBI Gene to PRO via UniProt and later extended to additionally use HomoloGene (Wheeler *et al.*, 2003) to account for genes for species outside the current scope of PRO (e.g. *Canis familiaris*). The resulting merged mapping can identify the relevant PRO identifier for approx. 95% of the NCBI Gene identifiers in the source data.

Annotations that are not associated with an identifier in one of the applied resources are stored in the mention-level data but not included in the entity-level graph. These annotations are thus included in document-level visualizations of annotations and accessible via various API functions, but cannot currently be part of discovery queries. In addition to the NCBI Gene identifiers that cannot be mapped to PRO, this filtering includes any mention in the source data that is not grounded to an external resource, including in particular many PubTator *Mutation* annotations (see Section 4.1).

### 3.3 Metrics and scoring functions

The LION LBD system design allows any number of edge weight metrics to be calculated over the graph and can switch between metrics on a query-by-query basis. The following edge weight metrics are currently implemented in the system: Co-occurrence count (*Count*) and Document count (*Doc-count*) are the number of sentences and documents (resp.) in which mentions of the entities connected by the edge co-occur. Jaccard index (*Jaccard*) is the ratio of the size of the intersection over the size of the union of the sets of sentences in which the entities occur. Symmetric conditional probability (*SCP*) is the product of the conditional probabilities of one entity being mentioned in a sentence where another occurs, and normalized pointwise mutual information (*NPMI*) is a measure of the independence of the mention occurrence distributions. Finally, Chi-squared ($\chi^{2}$), Student’s *t*-test (*t-test*) and log-likelihood ratio (*LLR*) are statistical tests measuring whether the mention distributions are independent of each other. We refer to the Supplementary Information for the detailed definitions of these metrics.

A number of alternatives for the scoring functions operating over the edge weights have also been implemented, and the choices between these can likewise be made independently for each query. For the aggregation function $f_{\text{g}}$, the alternatives *min*, *avg* and *max* are currently supported. As the names suggest, these functions assign as the score for a path the minimum, mean and maximum (resp.) of the edge weights on the path. For the accumulation function $f_{\text{c}}$, the choices *sum* and *max* are supported. When multiple paths lead to the same node, the former assigns as the node score the sum of the path scores while the latter takes the maximum of these scores.

We evaluated all combinations of metrics and scoring functions to determine the best system defaults (Section 4.2), and also offer users the option of selecting other configurations.

### 3.4 Database design

The differentiation between entities and their mentions (Section 2.2) is reflected in the two-level database design of the LION LBD system. Mention-level data is stored in a conventional SQL database. To support flexible and fast search of the entity graph, entity-level data is stored redundantly in a graph database and in a custom in-memory graph storage. For document texts and mention-level data we use PostgreSQL (https://www.postgresql.org/), an open source relational database supporting many advanced features such as full indexing for JSON data, which allows us to consistently use the flexible Web Annotation JSON-LD representation of annotations also in the database. The data of both mention and relation annotations is stored primarily in binary JSON columns, with performance-critical fields such as the ontology identifiers of related entities denormalized and separately indexed to directly support specific queries such as retrieving all sentences where entities with given ontology identifiers co-occur.

The entity and relation graph is stored in Neo4j (https://neo4j.com/), a specialized database available under open source licensing that supports native graph storage and querying using a custom graph-oriented query language. The use of Neo4j allows arbitrary queries over the graph to be expressed in a flexible and intuitive way. Finally, to support also the most demanding open discovery queries in a responsive manner, we have created a custom in-memory graph storage and search system specialized for LBD queries. This subsystem is implemented as a separate service with its own API and written in Python, with performance-critical sections in the Cython superset, which allows the generation of efficient C code.

### 3.5 Interfaces

**Fig. 4.**
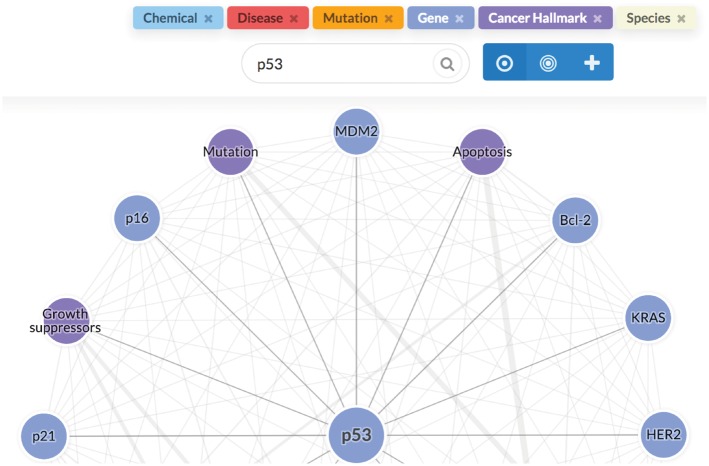
LION LBD user interface. The user query (*p53*) is shown together with controls switching between different discovery modes above the result graph, where nodes represent related concepts and edges their associations

To support direct browser-based use and programmatic access, we implement both a web-based user interface and an Application Programming Interface (API) to the LION LBD system, both implemented using the Python Flask (http://flask.pocoo.org) framework. The LION LBD web interface is responsive across devices, and is built on established web frameworks: we use Semantic UI (https://semantic-ui.com/) for layout and styling, and client-side functionality is implemented with jQuery. The Cytoscape.js library (Shannon *et al.*, 2003) is used for graph visualization, and Chart.js (http://www.chartjs.org/) for rendering charts. The API implements a conventional REST-like interface (Fielding and Taylor, 2000) using JSON for primary result data and Web Annotation JSON-LD for annotation data. Both the browser interface and the API are found along with detailed documentation at http://lbd.lionproject.net. Figure 4 illustrates the browser-based LION LBD UI.

### 3.6 Evaluation

LBD systems are commonly evaluated through the replication of previously published discoveries, most frequently using those reported by Swanson and colleagues over the years (e.g. Gordon and Lindsay, 1996, Weeber *et al.*, 2001). The basic approach is straightforward: the LBD system is set up to only use literature up to some cut-off date before the discovery and queried with terms relevant to the discovery (e.g. *migraine*); the system is successful if it identifies the reported associated concepts (e.g. *magnesium*). The performance of a system can be quantified e.g. by determining the rank of the expected response in the returned list of candidates (Srinivasan, 2004). Following previous work, we include the replication of Swanson’s discoveries as part of the evaluation of the LION LBD system. However, as the set of these discoveries is limited in size, somewhat dated and not directly relevant to cancer, we chose to also curate a new dataset of cancer-related discoveries from the literature for the primary evaluation of our proposed system.

To identify discoveries, we first surveyed articles published between 2006 and 2016 in journals that cover biomolecular cancer research (e.g. Science, Nature, The Lancet, British Journal of Cancer, and Cell). In the initial survey pass, we sought to identify specific cancer-related discoveries that can be characterized as a causal chain of three concepts (A-B-C). From the literature survey, we chose 50 candidate discoveries. In the second stage of processing, we filtered the candidates to identify discoveries that could have potentially been found by an LBD approach: namely, the two connections A-B and B-C should be found in the literature at some point in time before the connection between A and C is proposed. Specifically, we identified cases where in some year in the past, (A, B) and (B, C) each co-occur in at least 100 publications but are no (or very few) publications where (A, C) co-occur. This filtering was performed manually using PubMed searches, thus avoiding any possible bias toward specific NLP methods or LBD tools. Using this approach, we filtered the 50 candidates to 16. These candidates were then assessed by project participants to exclude connections that the domain experts judged to be too obvious (e.g. involving entities that are very well-studied in cancer biology) or insufficiently novel. This filtering selected a final set of 5 triples that represent specific recent discoveries on the molecular biology of cancer that could have potentially been suggested by an LBD system in the past. Finally, the relevant ontology and database identifier in the applied resources was manually identified for each of the concepts in the dataset. The dataset is presented in Table 1.

**Table 1.** Evaluation dataset from cancer research discoveries

A (id)	B (id)	C (id)	Reference
NF-*κ*B (PR:000001754)	Bcl-2 (PR:000002307)	Adenoma (MESH:D000236)	Van Der Heijden *et al.* (2016)
NOTCH1 (PR:000011331)	senescence (HOC:42)	C/EBP*β* (PR:000005308)	Hoare *et al.* (2016)
IL-17 (PR:000001138)	p38*α* (PR:000003107)	MKP-1 (PR:000006736)	Gaffen and McGeachy (2015)
Nrf2 (PR:000011170)	ROS (CHEBI:26523)	pancreatic cancer (MESH:D010190)	DeNicola *et al.* (2011)
CXCL12 (PR:000006066)	senescence (HOC:42)	thyroid cancer (MESH:D013964)	Kim *et al.* (2017)

We additionally evaluate the system using a set of Swanson’s discoveries. For each publication reporting such a discovery, we manually mapped the A and C terms to their associated database or ontology identifiers. We excluded the pair (*Fish oil*, *Raynaud’s syndrome*) Swanson (1986a) as the term *Fish oil* is not in scope for the entity types currently included in the LION LBD system. The evaluation dataset derived from Swanson’s discoveries is summarized in Table 2. We note that in addition to evaluation on confirmed biologically relevant discoveries, LBD systems can also be evaluated based on their ability to predict later edges based on earlier versions of the graph (Yetisgen-Yildiz and Pratt, 2009). We performed preliminary experiments using this type of time-slicing, but chose to forgo this setting due to the lack of ground truth in our data: there is no way to distinguish arbitrary co-occurrence from statements of biologically significant association.

**Table 2.** Evaluation dataset from Swanson’s discoveries

A (id)	C (id)	Reference
Migraine (MESH:D008881)	Magnesium (MESH:D008274)	Swanson (1988)
Somatomedin C (PR:000009182)	Arginine (CHEBI:29016)	Swanson (1990)
Alzheimer’s disease (MESH:D000544)	Estrogen (MESH:D004967)	Smalheiser and Swanson (1996b)
Alzheimer’s disease (MESH:D000544)	Indomethacin (MESH:D007213)	Smalheiser and Swanson (1996a)
Schizophrenia (MESH:D012559)	Calcium Independent Phospholipase A_2_ (PR:000012942)	Smalheiser and Swanson (1998)

To evaluate the system using these datasets, we consider all combinations of metrics and scoring functions (Section 3.3) and perform an open discovery query and a closed discovery query for each (A, B, C) triple (open discovery only for Swanson’s discoveries) using a version of the system data that only includes literature up to five years before the year of the relevant publication (Tables 1 and 2) and further excludes any document where A and C co-occur (Available from http://lbd.lionproject.net/downloads). In open discovery, we query the system for nodes indirectly connected with the A node and determine the rank of C in the results. In closed discovery, we query the system for nodes connecting A and C and identify the rank of B. This part of the evaluation does not assess the quality of responses differing from the expected one. We summarize the results over the different test cases by reporting the median rank of the expected node among the candidates retrieved by the system, which represents a typical number of responses a user would see before encountering the ‘correct’ response.

Finally, to assess the overall quality of the system responses, we manually analyze 100 candidates that were ranked higher than the expected response. This analysis is presented in Section 4.3.

## 4 Results and discussion

### 4.1 Data statistics

Key statistics for the mention and entity-level annotation are summarized in Table 3. The data covers nearly 170 M sentences in 27 M citations, and the overall density of annotation is high, averaging more than 1.5 entity mentions per sentence. Excepting for *Mutation*, tens of millions of mentions of each entity type are annotated. The fraction of all mentions that are grounded is high (82%), ranging 68–88% for types other than *Hallmark* and *Mutation.* For the former, all annotations are grounded by design; for the latter, only the SNP subset (16%) of PubTator *Mutation* annotations can be associated with an external database resource. The 217 M grounded mentions refer to 195 000 unique entities, thus averaging over 1000 mentions per entity. It is this difference in magnitude that allows the entity-level graph to be searched efficiently while representing all mentions in the literature: it would not be feasible to perform the queries over either a mention or unique string graph. The number of co-occurrence relations in the mention-level data is 258 133 610, and there are 12 797 488 edges representing these relations in the entity-level graph. The graph is thus densely connected, with an average of 66 neighbours per node, and highly connected nodes such as PR:000003035 (*p53*) have thousands of neighbours. As the neighbours of highly connected nodes also tend to be highly connected in a co-occurrence graph, open discovery searches starting from such nodes can visit a high fraction of the total number of nodes.

**Table 3.** Data statistics

Type	Mentions (Grounded)	Entities
Disease	81 993 034 (72 352 890)	9849
Chemical	68 839 682 (46 691 595)	110 024
Species	52 902 078 (45 937 366)	9765
Gene	31 545 993 (24 581 542)	27 089
Hallmark	26 769 779 (26 769 779)	37
Mutation	1 062 702 (174 531)	37 929

Total	263 113 268 (216 507 703)	194 693

### 4.2 Evaluation results

The results of the evaluation are summarized in Tables 4 and 5 for the cancer discoveries dataset and in Table 6 for Swanson’s discoveries. Full details for all are found in Supplementary Information. We first note that the system succeeds in recovering the expected term in all 15 cases, demonstrating its ability to replicate both discoveries used in previous LBD work and discoveries specifically relevant to cancer research. However, as the numbers of retrieved candidates can be large—here up to 425 in closed and 137 522 in open discovery—it is critical for usability that the system not only finds the target but also ranks it highly among the various candidates retrieved.

**Table 4.** Closed discovery evaluation results for cancer discoveries

Metric	Aggregation function $f_{\text{g}}$		
	min	avg	max
NPMI	86	**119**	**170**
SCP	70	196	299
$\chi^{2}$	74	196	270
*t*-test	**56**	136	261
LLR	65	163	264
Jaccard	81	213	282
Count	245	181	245
Doc-count	231	169	222

*Note*: Best result in each row underlined, best in column in bold.

**Table 5.** Open discovery evaluation results for cancer discoveries

Metric	Accumulation function $f_{\text{c}}$ (Aggregation function $f_{\text{g}}$**)**					
	sum (min)	max (min)	sum (avg)	max (avg)	sum (max)	max (max)
NPMI	98 698	15 476	121	5897	**36**	2268
SCP	276	926	400	1176	399	727
$\chi^{2}$	547	3582	402	1159	402	1159
*t*-test	118 751	**63**	98 406	325	125	176
LLR	98 677	187	344	646	319	645
Jaccard	29	1089	78	962	93	1122
Count	**15**	1005	**55**	**52**	62	**54**
Doc-count	23	738	72	68	74	68

*Note*: Best result in each row underlined, best in column in bold.

**Table 6.** Open discovery evaluation results for Swanson’s discoveries

Metric	Accumulation function $f_{\text{c}}$ (Aggregation function $f_{\text{g}}$**)**					
	sum (min)	max (min)	sum (avg)	max (avg)	sum (max)	max (max)
NPMI	41 837	8869	16 714	9715	74	5545
SCP	124	427	154	250	154	250
$\chi^{2}$	37 827	7820	156	263	155	263
*t*-test	40 103	1808	37 368	116	**5**	105
LLR	37 820	3404	9	45	10	**43**
Jaccard	**6**	1075	**6**	237	9	240
Count	8	43	20	**29**	21	261
Doc-count	7	**21**	20	31	21	237

*Note*: Best result in each row underlined, best in column in bold.

In closed discovery, the median rank ranges between 56 and 299 for the different parameterizations. The *min* aggregation function is preferred for all but the two basic count metrics (Count and Doc-count), which favor *avg* but perform below the more advanced edge weight metrics for the best aggregation functions. The best overall result is found for the *t*-test metric, and there is somewhat limited variation in results between this and the other non-count functions.

The evaluations using the open discovery task setting show much starker differences between different parameterizations, with the median rank of the target term ranging from 15 to 118 753 for cancer discoveries and 5 to 41 837 for Swanson’s discoveries. The *sum* accumulation function is preferred over *max* in all but two cases and produces the best overall results for both evaluation sets. For the aggregation function, *min* produces the best results in most cases, but both the *avg* and *max* alternatives are preferred for a number of metrics. By contrast to the closed discovery evaluation results, the basic count metrics perform quite competitively in open discovery, and results for other metrics are mixed: while SCP and Jaccard perform well for the *sum(min)* functions, many of the more advanced statistical metrics show very poor median ranks for this preferred combination. The best overall results are found for *sum(min(Count))* for the cancer discoveries and *sum(max(t-test))* for Swanson’s discoveries, with *sum(min(Jaccard))* as a close second.

Based on consistent and competitive performance in both settings and evaluation datasets, we chose to set *min* as the default aggregation function and *Jaccard* as the default metric in the system. In open discovery, the accumulation function is fixed to *sum* as it clearly outperformed the alternative (*max*). By contrast, we noted that the best results in individual cases were found for a variety of combinations of metric and aggregation function (see Supplementary Information), and have made the choice of these parameters available on the system interface to allow users to explore different combinations for their specific discovery tasks.

### 4.3 Manual analysis

While the above evaluation permits effective comparison of system variants, it is unrealistically demanding in only recognizing a single ‘correct’ target response for each case. In practice, a given A can be indirectly associated with several C concepts in open discovery, and there can be connections between A and C via more than one B in closed discovery.

To further quantify overall system performance and identify common sources of error, we manually analyzed ten system responses differing from the target response for each of the five cancer discovery cases in both open and closed discovery settings (i.e. 100 responses in total). To focus the analysis on cases that a user would be likely to see when using the system, we selected ten random responses ranking higher than the target response, or the ten highest-ranked responses excluding the target when the target was ranked in the top ten. The default metric and aggregation and accumulation functions were used in all cases.

In closed discovery, we found that 22 of the analyzed 50 responses (44%) represented potential connections between the given A and C, and in open discovery 17/50 (34%) were potential new indirectly connected concepts for the given A (the task setting and results are detailed in Supplementary Information). By far the most frequent type of error found in open discovery consisted of the system returning C terms that were judged to have a known direct connection with the given A (instead of a new, indirect connection), indicating that the graph lacks connections that are known or discoverable to human experts. Errors in closed discovery were more diverse, with common cases including unverifiable connections suggested by the system as well as instances where the flow of causality did not allow A-B-C chaining (e.g. A and C both affected B). These errors are likely due to the co-occurrence analysis generating spurious connections and only generating undirected edges in the graph.

The overall results of this analysis indicate that at least a third of the candidates suggested by the system are likely to be of potential interest to users, a result we consider very positive in the challenging LBD task. The analysis also suggests that system performance could be improved further through more extensive literature analysis (e.g. inclusion of full texts) and replacing co-occurrence analysis with a method that recognizes explicit causal statements and identifies connection directionality.

### 4.4 Case study

We demonstrate the use of the system by using it to assess whether arsenic increases the levels of the autotaxin (ATX) protein. By using the closed discovery mode in the LION tool, we entered search query arsenic as term A and autotaxin as term C. No direct connection was shown, which was expected as a search on PubMed using the combination of same two keywords (*arsenic* and *autotaxin*) gave no result. However, six intermediate genes were suggested by the LION LBD tool as appeared top ranked. The connection between arsenic—Nrf2—ATX was shown as having the strongest association. Nrf2 is a gene known to respond to oxidative stress and arsenic is an established inducer of oxidative stress, so this connection is in line with common knowledge and a search on PubMed for the terms *arsenic* and *Nrf2* gives 141 references. Searching *Nrf2* and *autotaxin* on PubMed gives only one reference (Venkatraman *et al.*, 2015). This reference shows that LPA (the product of ATX enzymatic activity) increases Nrf2 and antioxidant genes. Even though this information does not answer our question directly, it suggests a clear connection, possibly involving an altered redox signaling. This seemed credible, and might include an arsenic-induced ATX induction. Four other suggested genes (c-jun, MMP9, Rac1, cdc42) also gave references documenting connections to ATX in PubMed searches. AQP9 gave no references, but using ENPP2 (the ATX gene) gave one. Thus all genes suggested by the LION LBD tool were relevant. Furthermore, we tested this hypothesis in an experimental cell model and confirmed that arsenic induces ATX.

## 5 Conclusion

We have presented the LION LBD system for literature-based discovery focusing on molecular biology and cancer. The system is built using open data, open source and open standards, and is unique among available LBD systems in being based on state-of-the-art methods for named entity recognition and grounding and including references to the hallmarks of cancer in text. The LION LBD system offers both an interactive web-based interface for users and a programmable API. Evaluations of the system on cancer-related discoveries and Swanson’s discoveries demonstrated its ability to identify indirect connections and rank relevant concepts highly in both closed and open discovery settings. The system is presently limited to discovery over paths of length two and its source data to PubMed abstracts, and error analysis showed that the use of co-occurrence relations is a major factor affecting the quality of results. In the future, the LION LBD system will be maintained and further developed to address these and other limitations and to keep up to date with the most recent literature and advances in literature-based discovery. The system and all of its components are available under open licenses from the project home page http://lbd.lionproject.net.

## Funding

This work was supported by the Research Councils UK, grant number MR/M013049/1. MN lab is also supported by Cancer Research UK Cambridge Institute Core Grant (C14303/A17197).

*Conflict of Interest*: none declared.

## Supplementary Material

Supplementary Information
