# Supplementary material for: LION LBD: a literature-based discovery system for cancer biology
Source: Bioinformatics. 2018 Oct 9;35(9):1553–61. doi: 10.1093/bioinformatics/bty845 (PMC6499247; doi:10.1093/bioinformatics/bty845)
Supplement: Supplementary Information [file bty845_supplementary_information.pdf]

Supplementary Information for LION LBD: a Literature-Based Discovery System for Cancer Biology

This document contains supplementary information for the paper *LION LBD: a Literature-Based Discovery System for Cancer Biology.*

1 Task setting

For a simple weighted graph $G = (N, E)$ where N is the set of nodes, E the set of edges $E \subset N \times N$, $w(i, j)$ is the weight of the edge from i to j and f_{g} a weight aggregation function, the basic closed discovery task for nodes a and c can be addressed as

function ClosedDiscovery(a, c)
    for all b in $\mathcal{N}(a) \cap \mathcal{N}(c)$ **do**
        yield b, $f_{\mathrm{g}}(w(a, b), w(b, c))$

where $\mathcal{N}(i) = \{\, j \mid (i, j) \in E \,\}$ is the set of neighbours of node i and **yield** is defined as in Python as generating a sequence of returned values. Given an accumulation function f_{c} and a corresponding initial value s_0 (e.g. 0 for *sum* and $-\infty$ for *max*), open discovery can be similarly cast as

function OpenDiscovery(a)
    for all c in $\mathcal{N}(\mathcal{N}(a)) \setminus \mathcal{N}(a)$ **do**
        $s_c \leftarrow s_0$
    for all $b \in \mathcal{N}(a)$ **do**
        for all $c \in \mathcal{N}(b) \setminus \mathcal{N}(a)$ **do**
            $s_c \leftarrow f_{\mathrm{c}}(s_c, f_{\mathrm{g}}(w(a, b), w(b, c)))$
    for all c in $\mathcal{N}(\mathcal{N}(a)) \setminus \mathcal{N}(a)$ **do**
        yield c, s_c

As the relevant neighbour set operations can be implemented in linear time, the former algorithm has linear and the latter quadratic complexity with respect to the number of neighbours.[1]

[1] While fully sorting the returned (*node*, *score*) sequences requires $O(n \log n)$ time, the top k highest-scoring nodes can be determined in linear time, thus not impacting the overall complexity in either case.

2 Metrics

For terms a and b, we denote below the number of contexts where the terms (co-)occur by C and the total number of contexts by N. The *Count* and *Doc-count* metrics for an edge between nodes representing a and b are defined simply as $C(a,b)$ where the context is defined as a sentence for the former and a document for the latter. The context is set as sentence for all other metrics.

Pointwise mutual information (PMI) is defined as

$$w_{\text{PMI}}(a,b) = \log \frac{P(a,b)}{P(a)\,P(b)} = \log \frac{C(a,b)N}{C(a)C(b)} \tag{1}$$

and the normalized PMI (NPMI) metric as

$$w_{\text{NPMI}}(a,b) = \frac{w_{\text{PMI}}(a,b)}{-\log\left(P(a,b)\right)} = \frac{\log \frac{C(a,b)N}{C(a)C(b)}}{\log \frac{N}{C(a,b)}} \tag{2}$$

Symmetric conditional probability (SCP) is

$$w_{\text{SCP}}(a,b) = P(a|b)\,P(b|a) = \frac{P(a,b)^2}{P(a)\,P(b)} = \frac{C(a,b)^2}{C(a)C(b)} \tag{3}$$

and the Jaccard index as

$$w_{\text{Jaccard}}(a,b) = \frac{C(a,b)}{C(a)+C(b)-C(a,b)} \tag{4}$$

The Chi-squared (χ^2) metric is defined based on the contingency matrices of the observed and expected values as

$$w_{\chi^2} = \frac{N\left(C(a,b)\left(N+C(a,b)-C(a)-C(b)\right)-\left(C(a)-C(a,b)\right)\left(C(b)-C(a,b)\right)\right)^2}{C(a)C(b)\left(N-C(a)\right)\left(N-C(b)\right)} \tag{5}$$

and the Student's t-test statistic as

$$w_{t\text{-test}} = \frac{NC(a,b)-C(a)C(b)}{N\sqrt{C(a,b)}} \tag{6}$$

Finally, the log likelihood ratio (LLR) is estimated as

$$\begin{aligned} w_{\text{LLR}} = 2\Bigg(& C(a,b)\log\frac{C(a,b)N}{C(a)C(b)} + (C(b)-C(a,b))\log\frac{N(C(b)-C(a,b))}{C(b)(N-C(a))} \\ & + (C(a)-C(a,b))\log\frac{N(C(a)-C(a,b))}{C(a)(N-C(b))} \\ & + (N-C(a)-C(b)+C(a,b))\log\frac{N(N-C(a)-C(b)+C(a,b))}{(N-C(a))(N-C(b))}\Bigg) \end{aligned} \tag{7}$$

3 Results for cancer discoveries

Tables 1-5 present the detailed closed discovery results and Tables 6-10 the open discovery results for the five cancer discoveries test cases. In addition to the rank of the target concept among the returned results, we also report the total number of results (n) and the relative rank of the target. In all result tables, the best result in each row is underlined and the best result in each column in bold.

	Aggregation function f_g		
Metric	min	avg	max
NPMI	47 (2.00%)	<u>38</u> (1.61%)	120 (5.19%)
SCP	<u>10</u> (0.39%)	60 (2.57%)	65 (2.79%)
χ^2	<u>8</u> (0.31%)	60 (2.57%)	65 (2.79%)
t-test	<u>6</u> (0.22%)	**21** (0.87%)	**46** (1.96%)
LLR	<u>8</u> (0.31%)	47 (2.00%)	49 (2.09%)
Jaccard	<u>**4**</u> (0.13%)	34 (1.44%)	**46** (1.96%)
Count	<u>19</u> (0.78%)	47 (2.00%)	50 (2.14%)
Doc-count	<u>27</u> (1.13%)	49 (2.09%)	56 (2.40%)

Table 1: Closed discovery results for A=PR:000001754 (NF-κB), B=PR:000002307 (Bcl-2), C=MESH:D000236 (Adenoma), query year 2011, $n = 2294$.

	Aggregation function f_g		
Metric	min	avg	max
NPMI	504 (76.91%)	379 (57.80%)	<u>238</u> (36.24%)
SCP	564 (86.09%)	196 (29.82%)	<u>184</u> (27.98%)
χ^2	485 (74.01%)	196 (29.82%)	<u>184</u> (27.98%)
t-test	487 (74.31%)	275 (41.90%)	<u>**127**</u> (19.27%)
LLR	486 (74.16%)	**163** (24.77%)	<u>143</u> (21.71%)
Jaccard	515 (78.59%)	213 (32.42%)	<u>169</u> (25.69%)
Count	**429** (65.44%)	181 (27.52%)	<u>158</u> (24.01%)
Doc-count	508 (77.52%)	169 (25.69%)	<u>151</u> (22.94%)

Table 2: Closed discovery results for A=PR:000011331 (NOTCH1), B=HOC:42 (senescence), C=PR:000005308 (C/EBPβ), query year 2011, $n = 654$.

	Aggregation function f_g		
Metric	min	avg	max
NPMI	22 (4.94%)	<u>**3**</u> (0.47%)	**5** (0.94%)
SCP	<u>3</u> (0.47%)	5 (0.94%)	**5** (0.94%)
χ^2	<u>3</u> (0.47%)	5 (0.94%)	**5** (0.94%)
t-test	<u>6</u> (1.18%)	16 (3.53%)	21 (4.71%)
LLR	<u>5</u> (0.94%)	17 (3.76%)	17 (3.76%)
Jaccard	<u>**2**</u> (0.23%)	4 (0.71%)	**5** (0.94%)
Count	<u>12</u> (2.59%)	23 (5.18%)	23 (5.18%)
Doc-count	<u>12</u> (2.59%)	26 (5.88%)	27 (6.12%)

Table 3: Closed discovery results for A=PR:000001138 (IL-17), B=PR:000003107 (p38α), C=PR:000006736 (MKP-1), query year 2010, $n = 425$.

	Aggregation function f_g		
Metric	min	avg	max
NPMI	86 (19.14%)	**119** (26.58%)	**170** (38.06%)
SCP	70 (15.54%)	249 (55.86%)	284 (63.74%)
χ^2	74 (16.44%)	212 (47.52%)	224 (50.23%)
t-test	**56** (12.39%)	136 (30.41%)	237 (53.15%)
LLR	65 (14.41%)	203 (45.49%)	240 (53.83%)
Jaccard	81 (18.02%)	230 (51.58%)	253 (56.76%)
Count	245 (54.95%)	263 (59.01%)	258 (57.88%)
Doc-count	231 (51.80%)	329 (73.87%)	323 (72.52%)

Table 4: Closed discovery results for A=PR:000011170 (Nrf2), B=CHEBI:26523 (ROS), C=MESH:D010190 (pancreatic cancer), query year 2006, $n = 444$.

	Aggregation function f_g		
Metric	min	avg	max
NPMI	732 (69.69%)	824 (78.46%)	877 (83.51%)
SCP	614 (58.44%)	915 (87.13%)	955 (90.94%)
χ^2	771 (73.40%)	817 (77.79%)	871 (82.94%)
t-test	755 (71.88%)	786 (74.83%)	873 (83.13%)
LLR	766 (72.93%)	802 (76.36%)	871 (82.94%)
Jaccard	472 (44.90%)	813 (77.41%)	935 (89.04%)
Count	461 (43.85%)	734 (69.88%)	737 (70.16%)
Doc-count	**406** (38.61%)	**549** (52.24%)	**554** (52.72%)

Table 5: Closed discovery results for A=PR:000006066 (CXCL12), B=HOC:42 (senescence), C=MESH:D013964 (thyroid cancer), query year 2012, $n = 1049$.

	Accumulation function f_c (Aggregation function f_g)					
Metric	sum(min)	max(min)	sum(avg)	max(avg)	sum(max)	max(max)
NPMI	137498 (99.98%)	18377 (13.36%)	784 (0.57%)	24071 (17.50%)	**1** (0.00%)	7407 (5.39%)
SCP	42 (0.03%)	925 (0.67%)	336 (0.24%)	1286 (0.93%)	338 (0.25%)	1221 (0.89%)
χ^2	87183 (63.40%)	2241 (1.63%)	357 (0.26%)	1389 (1.01%)	357 (0.26%)	1198 (0.87%)
t-test	137514 (99.99%)	56 (0.04%)	137489 (99.98%)	**10** (0.01%)	2 (0.00%)	25 (0.02%)
LLR	137468 (99.96%)	169 (0.12%)	**1** (0.00%)	434 (0.31%)	**1** (0.00%)	441 (0.32%)
Jaccard	**1** (0.00%)	70 (0.05%)	**1** (0.00%)	1168 (0.85%)	**1** (0.00%)	1394 (1.01%)
Count	**1** (0.00%)	**19** (0.01%)	**1** (0.00%)	22 (0.01%)	2 (0.00%)	23 (0.02%)
Doc-count	**1** (0.00%)	60 (0.04%)	2 (0.00%)	19 (0.01%)	2 (0.00%)	**19** (0.01%)

Table 6: Open discovery results for A=PR:000001754 (NF-κB), C=MESH:D000236 (Adenoma), query year 2011, $n = 137522$.

	Accumulation function f_c (Aggregation function f_g)					
Metric	sum(min)	max(min)	sum(avg)	max(avg)	sum(max)	max(max)
NPMI	**10** (0.01%)	7887 (6.25%)	**7** (0.00%)	4817 (3.82%)	**20** (0.01%)	1400 (1.11%)
SCP	276 (0.22%)	2773 (2.20%)	400 (0.32%)	411 (0.32%)	399 (0.31%)	408 (0.32%)
χ^2	448 (0.35%)	3843 (3.04%)	402 (0.32%)	412 (0.33%)	402 (0.32%)	409 (0.32%)
t-test	118751 (94.11%)	**626** (0.50%)	19 (0.01%)	1252 (0.99%)	145 (0.11%)	1375 (1.09%)
LLR	57 (0.04%)	951 (0.75%)	382 (0.30%)	646 (0.51%)	472 (0.37%)	645 (0.51%)
Jaccard	13 (0.01%)	1991 (1.58%)	67 (0.05%)	**259** (0.20%)	93 (0.07%)	**258** (0.20%)
Count	382 (0.30%)	3285 (2.60%)	829 (0.66%)	3586 (2.84%)	933 (0.74%)	2393 (1.90%)
Doc-count	372 (0.29%)	3262 (2.58%)	942 (0.75%)	3292 (2.61%)	1088 (0.86%)	2736 (2.17%)

Table 7: Open discovery results for A=PR:000011331 (NOTCH1), C=PR:000005308 (C/EBPβ), query year 2011, $n = 126179$.

4 Results for Swanson's discoveries

Tables 11-15 present the detailed open discovery results for the Swanson's discoveries test cases. As for the cancer discoveries evaluation above, we report the rank of the target concept among

Metric	Accumulation function f_c (Aggregation function f_g)					
	sum(min)	max(min)	sum(avg)	max(avg)	sum(max)	max(max)
NPMI	**23** (0.02%)	4181 (3.52%)	27 (0.02%)	5897 (4.97%)	**149** (0.12%)	2268 (1.91%)
SCP	532 (0.45%)	6989 (5.89%)	658 (0.55%)	1176 (0.99%)	661 (0.56%)	**727** (0.61%)
χ^2	547 (0.46%)	4969 (4.18%)	652 (0.55%)	1159 (0.97%)	656 (0.55%)	1159 (0.97%)
t-test	105625 (88.96%)	1579 (1.33%)	**16** (0.01%)	1152 (0.97%)	289 (0.24%)	2386 (2.01%)
LLR	212 (0.18%)	1935 (1.63%)	469 (0.39%)	2907 (2.45%)	537 (0.45%)	6823 (5.75%)
Jaccard	135 (0.11%)	**1089** (0.92%)	353 (0.30%)	**962** (0.81%)	394 (0.33%)	1122 (0.94%)
Count	1435 (1.21%)	5495 (4.63%)	1124 (0.95%)	3709 (3.12%)	1053 (0.89%)	1860 (1.57%)
Doc-count	1566 (1.32%)	6235 (5.25%)	1291 (1.09%)	2815 (2.37%)	1232 (1.04%)	7481 (6.30%)

Table 8: Open discovery results for A=PR:000001138 (IL-17), C=PR:000006736 (MKP-1), query year 2010, $n = 118735$.

Metric	Accumulation function f_c (Aggregation function f_g)					
	sum(min)	max(min)	sum(avg)	max(avg)	sum(max)	max(max)
NPMI	98698 (99.75%)	27373 (27.66%)	121 (0.12%)	17420 (17.61%)	**36** (0.03%)	4992 (5.04%)
SCP	316 (0.32%)	926 (0.93%)	1034 (1.04%)	3709 (3.75%)	1039 (1.05%)	3638 (3.68%)
χ^2	98463 (99.51%)	3582 (3.62%)	1252 (1.26%)	3889 (3.93%)	1233 (1.25%)	3994 (4.04%)
t-test	98747 (99.80%)	**63** (0.06%)	98406 (99.45%)	325 (0.33%)	69 (0.07%)	176 (0.18%)
LLR	98677 (99.73%)	187 (0.19%)	344 (0.35%)	666 (0.67%)	319 (0.32%)	640 (0.65%)
Jaccard	268 (0.27%)	5062 (5.11%)	107 (0.11%)	2219 (2.24%)	104 (0.10%)	2451 (2.48%)
Count	**15** (0.01%)	514 (0.52%)	**55** (0.06%)	**49** (0.05%)	55 (0.06%)	**49** (0.05%)
Doc-count	23 (0.02%)	414 (0.42%)	56 (0.06%)	52 (0.05%)	57 (0.06%)	52 (0.05%)

Table 9: Open discovery results for A=PR:000011170 (Nrf2), C=MESH:D010190 (pancreatic cancer), query year 2006, $n = 98945$.

Metric	Accumulation function f_c (Aggregation function f_g)					
	sum(min)	max(min)	sum(avg)	max(avg)	sum(max)	max(max)
NPMI	132123 (99.71%)	15476 (11.68%)	612 (0.46%)	4568 (3.45%)	95 (0.07%)	1329 (1.00%)
SCP	58 (0.04%)	179 (0.13%)	341 (0.26%)	460 (0.35%)	343 (0.26%)	533 (0.40%)
χ^2	296 (0.22%)	223 (0.17%)	354 (0.27%)	757 (0.57%)	359 (0.27%)	588 (0.44%)
t-test	132397 (99.92%)	**4** (0.00%)	132356 (99.89%)	57 (0.04%)	125 (0.09%)	163 (0.12%)
LLR	132161 (99.74%)	5 (0.00%)	69 (0.05%)	406 (0.31%)	73 (0.05%)	805 (0.61%)
Jaccard	29 (0.02%)	497 (0.37%)	78 (0.06%)	155 (0.12%)	89 (0.07%)	705 (0.53%)
Count	**4** (0.00%)	1005 (0.76%)	**54** (0.04%)	**52** (0.04%)	**62** (0.05%)	**54** (0.04%)
Doc-count	10 (0.01%)	738 (0.56%)	72 (0.05%)	68 (0.05%)	74 (0.06%)	68 (0.05%)

Table 10: Open discovery results for A=PR:000006066 (CXCL12), C=MESH:D013964 (thyroid cancer), query year 2012, $n = 132503$.

the results, the total number of results (n), and the relative rank of the target. As above, the best result in each row is underlined and the best result in each column in bold in all result tables.

Metric	Accumulation function f_c (Aggregation function f_g)					
	sum(min)	max(min)	sum(avg)	max(avg)	sum(max)	max(max)
NPMI	37835 (99.85%)	8869 (23.41%)	790 (2.08%)	9229 (24.36%)	74 (0.20%)	1317 (3.48%)
SCP	327 (0.86%)	592 (1.56%)	70 (0.18%)	82 (0.22%)	71 (0.19%)	82 (0.22%)
χ^2	37827 (99.83%)	7820 (20.64%)	75 (0.20%)	84 (0.22%)	74 (0.20%)	84 (0.22%)
t-test	37822 (99.82%)	2517 (6.64%)	37368 (98.62%)	15 (0.04%)	18 (0.05%)	14 (0.04%)
LLR	37820 (99.81%)	3404 (8.98%)	**9** (0.02%)	**9** (0.02%)	**9** (0.02%)	**9** (0.02%)
Jaccard	51 (0.13%)	1728 (4.56%)	26 (0.07%)	66 (0.17%)	26 (0.07%)	66 (0.17%)
Count	16 (0.04%)	56 (0.15%)	20 (0.05%)	21 (0.06%)	21 (0.06%)	18 (0.05%)
Doc-count	**15** (0.04%)	**1** (0.00%)	20 (0.05%)	24 (0.06%)	21 (0.06%)	17 (0.04%)

Table 11: Open discovery results for A=MESH:D008881 (Migraine), C=MESH:D008274 (Magnesium), query year 1983, $n = 37892$.

	Accumulation function f_c (Aggregation function f_g)					
Metric	sum(min)	max(min)	sum(avg)	max(avg)	sum(max)	max(max)
NPMI	11 (0.03%)	267 (0.65%)	**1** (0.00%)	811 (1.97%)	**2** (0.00%)	514 (1.25%)
SCP	23 (0.06%)	31 (0.08%)	154 (0.37%)	200 (0.49%)	154 (0.37%)	199 (0.48%)
χ^2	29 (0.07%)	29 (0.07%)	156 (0.38%)	200 (0.49%)	155 (0.38%)	200 (0.49%)
t-test	40103 (97.50%)	56 (0.14%)	**1** (0.00%)	116 (0.28%)	3 (0.01%)	105 (0.26%)
LLR	36 (0.09%)	68 (0.17%)	5 (0.01%)	**45** (0.11%)	10 (0.02%)	**43** (0.10%)
Jaccard	**1** (0.00%)	**2** (0.00%)	5 (0.01%)	163 (0.40%)	6 (0.01%)	163 (0.40%)
Count	8 (0.02%)	43 (0.10%)	33 (0.08%)	318 (0.77%)	38 (0.09%)	261 (0.63%)
Doc-count	3 (0.01%)	21 (0.05%)	33 (0.08%)	313 (0.76%)	38 (0.09%)	237 (0.58%)

Table 12: Open discovery results for A=PR:000009182 (Somatomedin C), C=CHEBI:29016 (Arginine), query year 1985, $n = 41131$.

	Accumulation function f_c (Aggregation function f_g)					
Metric	sum(min)	max(min)	sum(avg)	max(avg)	sum(max)	max(max)
NPMI	58865 (100.00%)	12379 (21.03%)	58864 (100.00%)	17449 (29.64%)	174 (0.30%)	7071 (12.01%)
SCP	18 (0.03%)	28 (0.05%)	52 (0.09%)	250 (0.42%)	52 (0.09%)	250 (0.42%)
χ^2	58865 (100.00%)	987 (1.68%)	54 (0.09%)	263 (0.45%)	54 (0.09%)	263 (0.45%)
t-test	58865 (100.00%)	51 (0.09%)	58865 (100.00%)	**1** (0.00%)	5 (0.01%)	**1** (0.00%)
LLR	58865 (100.00%)	101 (0.17%)	**1** (0.00%)	**1** (0.00%)	**1** (0.00%)	**1** (0.00%)
Jaccard	6 (0.01%)	461 (0.78%)	2 (0.00%)	237 (0.40%)	2 (0.00%)	240 (0.41%)
Count	**2** (0.00%)	3 (0.01%)	**1** (0.00%)	**1** (0.00%)	**1** (0.00%)	**1** (0.00%)
Doc-count	3 (0.01%)	**1** (0.00%)	**1** (0.00%)	**1** (0.00%)	**1** (0.00%)	**1** (0.00%)

Table 13: Open discovery results for A=MESH:D000544 (Alzheimer's disease), C=MESH:D004967 (Estrogen), query year 1991, $n = 58866$.

	Accumulation function f_c (Aggregation function f_g)					
Metric	sum(min)	max(min)	sum(avg)	max(avg)	sum(max)	max(max)
NPMI	58857 (99.99%)	8629 (14.66%)	58840 (99.96%)	9715 (16.50%)	30 (0.05%)	5545 (9.42%)
SCP	124 (0.21%)	427 (0.73%)	405 (0.69%)	1633 (2.77%)	405 (0.69%)	1631 (2.77%)
χ^2	58857 (99.99%)	13013 (22.11%)	457 (0.78%)	1646 (2.80%)	452 (0.77%)	1644 (2.79%)
t-test	58854 (99.99%)	1808 (3.07%)	58846 (99.97%)	25666 (43.60%)	**1** (0.00%)	23966 (40.72%)
LLR	58854 (99.99%)	3407 (5.79%)	20 (0.03%)	76 (0.13%)	20 (0.03%)	**70** (0.12%)
Jaccard	**6** (0.01%)	1075 (1.83%)	6 (0.01%)	994 (1.69%)	9 (0.02%)	993 (1.69%)
Count	8 (0.01%)	**28** (0.05%)	6 (0.01%)	**29** (0.05%)	6 (0.01%)	24072 (40.90%)
Doc-count	7 (0.01%)	31 (0.05%)	**5** (0.01%)	31 (0.05%)	5 (0.01%)	24170 (41.06%)

Table 14: Open discovery results for A=MESH:D000544 (Alzheimer's disease), C=MESH:D007213 (Indomethacin), query year 1991, $n = 58862$.

	Accumulation function f_c (Aggregation function f_g)					
Metric	sum(min)	max(min)	sum(avg)	max(avg)	sum(max)	max(max)
NPMI	41837 (65.89%)	33884 (53.37%)	16714 (26.32%)	29246 (46.06%)	22122 (34.84%)	38396 (60.47%)
SCP	22002 (34.65%)	37252 (58.67%)	25093 (39.52%)	12955 (20.40%)	25192 (39.68%)	11340 (17.86%)
χ^2	33790 (53.22%)	30165 (47.51%)	25171 (39.64%)	21321 (33.58%)	25863 (40.73%)	16825 (26.50%)
t-test	35556 (56.00%)	30949 (48.74%)	31641 (49.83%)	14148 (22.28%)	**19755** (31.11%)	9695 (15.27%)
LLR	31629 (49.82%)	26828 (42.25%)	**16398** (25.83%)	13184 (20.76%)	21797 (34.33%)	13332 (21.00%)
Jaccard	**15834** (24.94%)	14880 (23.44%)	21675 (34.14%)	19067 (30.03%)	22233 (35.02%)	17932 (28.24%)
Count	17387 (27.38%)	**14281** (22.49%)	21150 (33.31%)	10711 (16.87%)	21260 (33.48%)	2470 (3.89%)
Doc-count	17326 (27.29%)	14301 (22.52%)	21089 (33.22%)	**10630** (16.74%)	21213 (33.41%)	**2401** (3.78%)

Table 15: Open discovery results for A=MESH:D012559 (Schizophrenia), C=PR:000012942 (Calcium Independent Phospholipase A_2), query year 1993, $n = 63492$.

5 Manual analysis

This section details the task setting and results of the manual analysis.

Each case is defined by a query year and either an (A,C) term pair (closed discovery) or an A term (open discovery). In closed discovery, the annotators were provided with B terms and asked to decide for each *is there a potential biologically meaningful connection between A and C via B.* In open discovery, annotators were provided with C terms and asked to decide for each *is there a potential biologically meaningful indirect connection between A and C.*

The annotators were asked to consider each case in the context of its query year (i.e. reflecting the state of published knowledge at the time) and record their judgment for each candidate simply as “yes” or “no”. For open discovery, annotators were additionally instructed to answer “no” for any known *direct* connections between the query term A and any candidate C.

The candidates and annotator judgments are shown in Tables 16-20 for closed discovery and Tables 21-25 for open discovery.

B ID	B name	judgment
PR:000025365	cytochrome c oxidase subunit 2	no
MESH:D003110	Colonic Neoplasms	no
PR:000003035	cellular tumor antigen p53	yes
PR:000013428	prostaglandin G/H synthase 2	yes
PR:000005121	G1/S-specific cyclin-D1	yes
PR:000002198	catenin beta-1	yes
MESH:D010190	Pancreatic Neoplasms	no
HOC:92	Growth promoting signals	no
HOC:9	Proliferative signaling	no
MESH:D009369	Neoplasms	no

Table 16: Closed discovery analysis for A=PR:000001754 (NF-κB), C=MESH:D000236 (Adenoma), query year 2011.

B ID	B name	judgment
PR:000007106	endothelial PAS domain-containing protein 1	no
PR:000011152	neurogenic differentiation factor 1	no
PR:000001412	CD86 molecule	no
PR:000026269	survival motor neuron protein	no
PR:000003809	alpha-fetoprotein	yes
PR:000015058	solute carrier family 2, facilitated glucose transporter member 1	no
MESH:D006528	Carcinoma, Hepatocellular	no
PR:000006066	stromal cell-derived factor 1	yes
PR:000012332	histone acetyltransferase KAT2B	yes
PR:000001393	interleukin-6	yes

Table 17: Closed discovery analysis for A=PR:000011331 (NOTCH1), C=PR:000005308 (C/EBPβ), query year 2011.

B ID	B name	judgment
MESH:C001899	triptolide	no
PR:000010413	macrophage migration inhibitory factor	no
PR:000002206	mitogen-activated protein kinase 8	yes
PR:000002121	C-C motif chemokine 13	no
PR:000005308	CCAAT/enhancer-binding protein beta	yes
PR:000005145	cyclin-dependent kinase 20	no
PR:000006063	growth-regulated alpha protein	yes
MESH:C093642	SB 203580	no
PR:000010902	mast cell protease 1	no
PR:000010488	stromelysin-1	no

Table 18: Closed discovery analysis for A=PR:000001138 (IL-17), C=PR:000006736 (MKP-1), query year 2010.

B ID	B name	judgment
MESH:D064420	Drug-Related Side Effects and Adverse Reactions	no
PR:000017049	UDP-glucuronosyltransferase 1-10	no
MESH:D005978	Glutathione	yes
PR:000000103	mitogen-activated protein kinase 1	yes
PR:000001962	tumor necrosis factor receptor superfamily member 6	yes
MESH:D009393	Nephritis	no
PR:000007597	proto-oncogene c-Fos	yes
PR:000014413	protein S100-A6	yes
PR:000001970	vascular cell adhesion protein 1	yes
PR:000007498	fibroblast growth factor 7	no

Table 19: Closed discovery analysis for A=PR:000011170 (Nrf2), C=MESH:D010190 (pancreatic cancer), query year 2006.

B ID	B name	judgment
CHEBI:61432	phosphotungstic acid polymer	no
MESH:D006331	Heart Diseases	no
MESH:D015470	Leukemia, Myeloid, Acute	no
PR:000017445	protein Wnt-5a	yes
MESH:D003111	Colonic Polyps	no
MESH:C012589	trichostatin A	yes
PR:000001850	cathepsin K	yes
PR:000002193	apoptosis regulator BAX	yes
MESH:C471405	sorafenib	yes
MESH:C562463	Pancreatic Carcinoma	no

Table 20: Closed discovery analysis for A=PR:000006066 (CXCL12), C=MESH:D013964 (thyroid cancer), query year 2012.

C ID	C name	judgment
MESH:D002869	Chromosome Aberrations	no
PR:000011387	neuropeptide Y	no
PR:000004900	complement C3	no
MESH:D019342	Acetic Acid	no
MESH:D002331	Carnitine	no
CHEBI:16027	adenosine 5'-monophosphate	no
PR:000001843	Thy-1 membrane glycoprotein	yes
MESH:D009503	Neutropenia	no
NCBITaxon:3847	Glycine max	no
MESH:D013921	Thrombocytopenia	yes

Table 21: Open discovery analysis for A=PR:000001754 (NF-κB), query year 2011.

C ID	C name	judgment
PR:000002035	beta-type platelet-derived growth factor receptor	no
PR:000002317	caspase-8	yes
PR:000000364	smad2	no
MESH:C113580	U 0126	no
PR:000000176	GTP-binding protein RhoA	no
MESH:D053632	X-Linked Combined Immunodeficiency Diseases	no
PR:000010125	dual specificity mitogen-activated protein kinase kinase 1	yes
PR:000008902	interferon alpha-inducible protein 27	yes
PR:000006937	early growth response protein 1	no
MESH:D020151	Protein C Deficiency	no

Table 22: Open discovery analysis for A=PR:000011331 (NOTCH1), query year 2011.

C ID	C name	judgment
PR:000002309	caspase-1	no
PR:000012289	poly [ADP-ribose] polymerase 1	yes
MESH:D000241	Adenosine	no
NCBITaxon:9615	Canis lupus familiaris	no
PR:000009054	insulin	no
PR:000001465	high affinity immunoglobulin gamma Fc receptor I	yes
CHEBI:35366	fatty acid	no
PR:000024839	heat shock 70 kDa protein 1B	yes
PR:000002998	tyrosine-protein kinase Lck	yes
CHEBI:52450	2-O-acetyl-1-O-octadecyl-sn-glycero-3-phosphocholine	no

Table 23: Open discovery analysis for A=PR:000001138 (IL-17), query year 2010.

C ID	C name	judgment
PR:000002112	vascular endothelial growth factor receptor 2	yes
MESH:D008545	Melanoma	yes
MESH:D000244	Adenosine Diphosphate	no
PR:000007840	glyceraldehyde-3-phosphate dehydrogenase	yes
MESH:C038491	allyl sulfide	no
PR:000007640	forkhead box protein O1	yes
MESH:D011064	Poly Adenosine Diphosphate Ribose	no
PR:000000182	TGF-beta 1	no
MESH:D017239	Paclitaxel	yes
PR:000006130	cytochrome P450 3A4	no

Table 24: Open discovery analysis for A=PR:000011170 (Nrf2), query year 2006.

C ID	C name	judgment
MESH:D012175	Retinoblastoma	no
PR:000001945	transferrin receptor protein 1	no
PR:000001153	Toll-like receptor 2	yes
PR:000003414	heat shock 70 kDa protein 4	no
PR:000016285	protransforming growth factor alpha	yes
MESH:D018235	Smooth Muscle Tumor	no
CHEBI:4806	(-)-epigallocatechin 3-gallate	no
PR:000001905	platelet glycoprotein 4	yes
PR:000001083	CD2 molecule	no
MESH:C059514	resveratrol	no

Table 25: Open discovery analysis for A=PR:000006066 (CXCL12), query year 2012.
